# Reliability and Validity of the Chinese Version of the Premenstrual Symptoms Screening Tool

**DOI:** 10.1002/brb3.71455

**Published:** 2026-04-30

**Authors:** Xue Li, Xiaochun Wang

**Affiliations:** ^1^ School of Psychology Shanghai University of Sport Shanghai China

**Keywords:** Chinese version, female college students, PSST, reliability, validity

## Abstract

**Objective:**

To evaluate the reliability and validity of the Chinese version of the Premenstrual Symptoms Screening Tool (PSST) in a sample of Chinese female college students.

**Methods:**

A total of 1366 female college students completed the Chinese version of the PSST, the Patient Health Questionnaire (PHQ), and the Generalized Anxiety Disorder Scale (GAD). According to the screening criteria of the PSST, 196 participants were categorized into the premenstrual syndrome (PMS) group, 24 participants were categorized into the premenstrual dysphoric disorder (PMDD) group, and the remaining 1146 were assigned to the healthy control group. For test–retest reliability assessment, a separate sample of 50 female college students completed the Chinese PSST twice, with a retest interval of 28–32 days (*M* = 30.27, SD = 0.55).

**Results:**

The Cronbach's *α* coefficients for the Symptom Dimension, Impact Dimension, and total scale of the Chinese PSST were 0.95, 0.93, and 0.91, respectively; the corresponding test–retest reliability coefficients (intraclass correlation coefficient [ICC]) were 0.89, 0.99, and 0.94, respectively. The scale showed satisfactory empirical validity: the proportion of moderate‐to‐severe symptom endorsements (score = 2–3) across all PSST items was significantly higher in the PMS group and the PMDD group than in the healthy control group (all *p* < 0.001). Scores on the total PSST and its two dimensions were significantly positively correlated with scores on the PHQ and GAD (all *p* < 0.001). For construct validity, confirmatory factor analysis (CFA) showed that the Chinese PSST had a two‐factor structure (Symptom Dimension and Impact Dimension) with acceptable model fit indices (*χ* ^2^/df = 4.21, GFI = 0.932, CFI = 0.925, TLI = 0.905, RMSEA = 0.05), indicating good construct validity.

**Conclusion:**

The Chinese version of the PSST exhibits excellent reliability and validity among Chinese female college students and thus serves as a reliable tool for screening moderate‐to‐severe PMS in this population.

## Graphical abstract

The Chinese PSST was validated in 1366 female college students. It showed excellent reliability and validity, effectively distinguishing PMS/PMDD from healthy controls with a stable two‐factor structure.

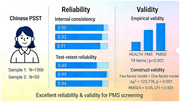

## 1 Introduction

Premenstrual syndrome (PMS) is a clinical syndrome characterized by cyclic emotional and physical symptoms that occur in the luteal phase of the menstrual cycle and resolve shortly after menstruation onset. Emotionally, core manifestations include depression, anxiety, and irritability; physically, symptoms typically involve breast tenderness, lower abdominal distension and pain, headaches, and limb swelling (Dueñas et al. 2011). Premenstrual dysphoric disorder (PMDD) represents a severe, clinically significant subtype of PMS, associated with more intense symptoms that substantially impair daily functioning (Lustyk et al. 2009).

A variety of screening tools and scales have been developed for the assessment of PMS, including the Premenstrual Symptoms Screening Tool (PSST) (Steiner et al. 2003), the Premenstrual Syndrome‐Total Symptom Questionnaire (PMS‐TSQ) (Endicott et al. 1995), the Daily Record of Severity of Problems (DRSP) (Endicott et al. 1996), and the Premenstrual Assessment Form (PAF) (Halbreich et al. 1982). However, these tools exhibit notable limitations that hinder their widespread applicability: The PMS‐TSQ lacks a standardized positive cutoff value, with reported thresholds varying widely across studies (ranging from 30 to 50 points). This variability severely compromises the comparability of results between different research projects. The DRSP requires continuous daily recording over at least two complete menstrual cycles, a protocol that is operationally cumbersome. This complexity not only leads to high participant dropout rates and low efficiency in clinical settings but also creates a significant barrier to adoption by nonpsychiatric healthcare providers. The PAF comprises 95 items, requiring approximately 20 min to complete. Its length easily induces respondent fatigue, reducing data quality. Furthermore, its symptom items are primarily tailored to the clinical manifestations of Western populations, resulting in limited cultural adaptability to Eastern populations. When applied to young demographic groups (e.g., college students), it also demonstrates poor dimensional discriminant validity. In contrast, the PSST combines practicality and scientific rigor, addressing the limitations of existing scales. It features concise content (19 items total), requires only 5–8 min to complete, and adopts a retrospective self‐report format that eliminates the need for long‐term daily recording. This design significantly improves participant compliance, making it well‐suited for large‐scale population screening and rapid clinical assessment. Notably, its dimensional framework—centered on “symptom manifestations + functional impact”—not only comprehensively covers the core symptoms specified in the Diagnostic and Statistical Manual of Mental Disorders (DSM) diagnostic criteria but also quantifies the interference of symptoms with work, family, and social functioning. This dual focus aligns closely with the practical demands of clinical practice, enhancing its utility for both research and clinical settings.

Despite these advantages, publicly available data on the reliability and validity of the Chinese version of the PSST remain limited—particularly when applied to college student populations, which possess unique physiological and psychological characteristics. Therefore, the primary objective of this study is to systematically evaluate and report the reliability and validity of the Chinese version of the PSST among Chinese college students, thereby providing a psychometrically sound assessment tool for PMS research in this demographic.

## 2 Participants and Methods

### 2.1 Participants

#### 2.1.1 Sample 1

A convenience sampling approach was employed to recruit 1500 female students (encompassing both undergraduate and master's degree candidates) from six universities located in Shanghai, China. To ensure data quality, questionnaires were excluded if they met any of the following criteria: incomplete responses, identical answers across all items, obviously patterned and presumably falsified responses, irregular menstrual cycles, physical illnesses, or the use of hormonal medications. After this screening process, 1366 valid questionnaires were retained, resulting in an effective response rate of 91.07%. The age range of the participants was 18–32 years, with a mean age of 19.74 years (standard deviation [SD] = 2.11) and a median age of 19.00 years.

#### 2.1.2 Sample 2

Convenience sampling was also used to select 60 female college students from a single university in Shanghai. Among these participants, 50 completed both rounds of testing in full, leading to an effective participation rate of 83.33%. The participants were aged between 19 and 20 years, with a mean age of 19.14 years (SD = 0.35). The interval between the second and first test administrations ranged from 28 to 32 days, with an average interval of 30.27 days (SD = 0.55).

### 2.2 Instruments

#### 2.2.1 PSST

The PSST is a self‐report instrument designed to screen for both PMS and PMDD, addressing the full spectrum of premenstrual disorders (Steiner et al. 2003). The Chinese version of the PSST used in this study was translated by researchers from Nanjing University. The PSST comprises 19 items, which are divided into two dimensions—Symptom Dimension: The first 14 items assess the severity of premenstrual symptoms in female participants; Impact Dimension: The final five items evaluate the influence of these symptoms (if present) on multiple domains of participants’ lives, including family relationships, work performance, and other relevant aspects.

Responses to the questionnaire are rated on a 4‐point Likert scale, ranging from 0 (*not at all*) to 3 (*very severe*). Higher total scores indicate either more severe premenstrual symptoms or greater life impact caused by these symptoms. For participant classification, the screening criteria proposed by the scale's original authors were adopted, as follows:

PMS: A participant is identified as having PMS if they meet all three of the following conditions: (1) at least one item among Items 1–4 receives a score of 2; (2) at least four of the remaining items in Items 1–14 (excluding the item that meets the first condition) receive a score of 2; and (3) at least one item among Items 15–19 receives a score of 2.

PMDD: A participant is identified as having PMDD if they meet all three of the following conditions: (1) at least one item among Items 1–4 receives a score of 3; (2) at least four of the remaining items in Items 1–14 (excluding the item that meets the first condition) receive a score of 2; and (3) at least one item among Items 15–19 receives a score of 3.

#### 2.2.2 Patient Health Questionnaire (PHQ)

The PHQ is a widely used self‐report instrument for screening and assessing depressive symptoms, developed by Kroenke et al. (2001). Comprising nine items, the scale evaluates the frequency of core depressive symptoms (e.g., anhedonia, low mood, sleep disturbances, fatigue, and suicidal ideation) experienced over the past 2 weeks.

Responses are rated on a 4‐point Likert scale, ranging from 0 (*not at all*) to 3 (*nearly every day*). Total scores range from 0 to 27, with higher scores indicating more frequent and severe depressive symptoms. Clinically validated cutoff values for the PHQ include 0–4 points (no depressive symptoms), 5–9 points (mild depression), 10–14 points (moderate depression), 15–19 points (moderately severe depression), and 20–27 points (severe depression)

The Chinese revised version of the PHQ has demonstrated excellent internal consistency, with a Cronbach's *α* coefficient of 0.898 in previous validation studies (Y. L. Zhang and Liu 2006), consistent with the scale's strong reliability in international populations (Cronbach's *α* = 0.86–0.89).

#### 2.2.3 Generalized Anxiety Disorder Scale‐7 (GAD)

The GAD is a brief self‐report tool designed to screen for generalized anxiety disorder, developed by Spitzer et al. (2006). While the original scale consists of seven items assessing key anxiety symptoms (e.g., excessive worry, restlessness, irritability, and difficulty relaxing), the seven‐item version utilized in this study retains core content related to anxiety frequency over the past 2 weeks.

Similar to the PHQ, responses are scored on a 4‐point Likert scale (0 = *not at all* to 3 = *nearly every day*). Total scores correlate with anxiety severity: 0–4 points (no anxiety), 5–9 points (mild anxiety), 10–14 points (moderate anxiety), and ≥15 points (severe anxiety), with scores ≥10 indicating clinically significant anxiety requiring further evaluation.

The Chinese revised version of the GAD has shown good psychometric properties, with a Cronbach's *α* coefficient of 0.85 (He et al. 2010), supporting its reliability for use in Chinese populations.

### 2.3 Research Procedure

A total of 1500 female college students were recruited from six universities and 305 classes in Shanghai, China. The survey was uniformly administered by class counselors during class time. Counselors introduced the purpose and background of the survey and displayed the survey QR code, and willing participants completed the online questionnaire by scanning the code on‐site. Prior to participation, all participants provided verbal informed consent, during which they were fully informed of the study's purpose, data usage, and their right to withdraw at any time without penalty.

A standardized set of instructions was developed to ensure consistency in questionnaire administration. The instructions detailed key information, including (1) the confidentiality of all responses (with data anonymized and used solely for research purposes); (2) the requirement to provide truthful answers to ensure data validity; (3) precautions for completion (e.g., avoiding hasty responses); and (4) step‐by‐step guidance on the filling method.

For Sample 1, participants completed three instruments: the Chinese version of the PSST, the PHQ, and the GAD. The total completion time for these questionnaires was approximately 8 min. For Sample 2, participants completed the Chinese version of the PSST twice (consistent with the test–retest design). Each administration of the PSST took approximately 5 min, with the interval between the two tests ranging from 28 to 32 days (*M* = 30.27, SD = 0.55), as previously reported in Section 2.1.

### 2.4 Data Analysis

All statistical analyses were conducted using professional software, with specific tools selected based on the type of psychometric indicator being evaluated.

SPSS 27.0 was used for the following analyses: descriptive statistics of demographic characteristics (e.g., age, height, weight, menstrual cycle) for all participants; internal consistency reliability of the PSST (assessed via Cronbach's *α* coefficient); test–retest reliability of the PSST (evaluated using the intraclass correlation coefficient [ICC] to measure consistency between the two administrations in Sample 2); empirical validity of the PSST (examined by analyzing the distribution of PMS screening results and their intergroup differences); and criterion validity of the PSST (assessed via Pearson correlation analysis between PSST scores and scores on the PHQ and GAD, as indicators of depressive and anxiety symptoms).

AMOS 27.0 was employed to verify the construct validity of the PSST. Specifically, a confirmatory factor analysis (CFA) was conducted to test the fit of the proposed two‐dimensional structure (Symptom Dimension and Impact Dimension) to the data from Sample 1.

To ensure clarity in data usage, analyses were stratified by sample type: data from Sample 1 (*n* = 1366) were used for demographic characteristic analysis, internal consistency reliability, empirical validity, criterion validity, and construct validity; data from Sample 2 (*n* = 50) were exclusively utilized to assess test–retest reliability, given its repeated‐measures design.

## 3 Results

### 3.1 Demographic Characteristics

Based on the screening criteria of the PSST, 196 participants in Sample 1 were identified as having PMS and 24 as having PMDD; the remaining 1146 participants were categorized as healthy controls. The detection rate of PMS was approximately 14.35%, and the detection rate of PMDD was approximately 1.76%. Demographic characteristics of the PMS group, the PMDD group, and the healthy control group are summarized in Table 1. Independent samples *t*‐tests revealed no statistically significant differences among the three groups for age (*t*(1364) = 0.849, *p* > 0.05), height (*t*(1364) = 0.694, *p* > 0.05), or weight (*t*(1364) = 0.121, *p* > 0.05).

**TABLE 1:** Demographic characteristics of the PMS group and the healthy control group (*M* ± SD).

	Overall (*n* = 1366)	PMS group (*n* = 196)	PMDD group (*n* = 24)	Healthy control group (*n* = 1146)
Age	19.74 ± 2.11	19.82 ± 2.17	20.00 ± 2.64	19.71 ± 2.08
Height	164.51 ± 5.91	164.48 ± 5.79	167.08 ± 7.88	164.46 ± 5.87
Weight	54.58 ± 7.49	54.38 ± 7.00	56.73 ± 7.83	54.57 ± 7.56

### 3.2 Reliability

#### 3.2.1 Internal Consistency Reliability

Internal consistency reliability of the Chinese version of the PSST was assessed using Cronbach's *α* coefficient, with data derived from Sample 1. Results showed that the overall Cronbach's *α* coefficient for college students was 0.95. Specifically, the *α* coefficients for the Symptom Dimension subscale and Impact Dimension subscale were 0.93 and 0.91, respectively.

#### 3.2.2 Test–Retest Reliability

Test–retest reliability of the PSST was evaluated via correlation analysis of scores across the two administrations, using data from Sample 2. Results indicated that the test–retest reliability coefficient for the total scale was 0.94 (*p* < 0.001). For the subscales, the test–retest reliability coefficients were 0.89 for the Symptom Dimension and 0.99 for the Impact Dimension, with both values being statistically significant (both *p* < 0.001).

### 3.3 Validity

#### 3.3.1 Empirical Validity

Consistent with the methodological protocols used in the development and revision of the original PSST, the proportions of participants in the PMS group, the PMDD group, and the healthy control group who endorsed “moderate” (score = 2) or “very severe” (score = 3) for each item were computed. Chi‐square (*χ* ^2^) tests were conducted to compare these response proportions among the three groups, with results summarized in Table 2.

**TABLE 2:** Proportions of participants endorsing moderate‐to‐severe responses for each PSST item between the PMS group, the PMDD group, and the healthy control group.

Item	PMS group (*n* = 196)	PMDD group (*n* = 24)	Healthy control group (*n* = 1146)	*χ* ^2^
1. Anger/irritability	61.22%	83.33%	9.77%	291.29^***^
2. Anxiety/tension	57.14%	83.33%	6.20%	262.11
3. Tearful/increased sensitivity to rejection	76.53%	91.67%	16.32%	352.00^***^
4. Decreased mood/hopelessness	76.53%	95.83%	9.25%	389.02^***^
5. Decreased interest in work activities	78.57%	87.50%	14.14%	370.05^***^
6. Decreased interest in home activities	61.73%	58.33%	5.93%	225.07^***^
7. Decreased interest in social activities	67.86%	79.17%	8.73%	287.06^***^
8. Difficulty concentrating	62.76%	75.00%	7.68%	260.01^***^
9. Fatigue/lack of energy	85.71%	95.83%	32.98%	428.11^***^
10. Overeating/food cravings	68.88%	58.33%	20.77%	249.08^***^
11. Insomnia	34.18%	25.00%	4.10%	110.05^***^
12. Hypersomnia	69.90%	83.33%	18.41%	319.15^***^
13. Feeling overwhelmed or out of control	51.53%	87.50%	3.23%	265.09^***^
14. Physical symptoms: breast tenderness, headaches, joint/muscle pain, bloating, weight gain	71.94%	75.00%	20.24%	302.12^***^
15. The above (1–14) symptoms affect your work efficiency or productivity	85.20%	95.83%	6.37%	417.03^***^
16. The above (1–14) symptoms affect your relationships with coworkers	43.88%	66.67%	1.57%	168.04^***^
17. The above (1–14) symptoms affect your relationships with your family	42.86%	45.83%	1.40%	102.03^***^
18. The above (1–14) symptoms affect your social life activities	58.16%	79.17%	2.62%	228.06^***^
19. The above (1–14) symptoms affect your home responsibilities	34.18%	41.67%	1.22%	91.07^***^

***p < 0.001.

These analyses revealed statistically significant differences among the PMS group, the PMDD group, and the healthy control group in the proportions of “moderate” or “very severe” responses across all items (all *p* < 0.001). Specifically, the PMDD group consistently demonstrated higher proportions of such endorsements compared to the PMS group and the healthy control group.

#### 3.3.2 Criterion Validity

Independent samples *t*‐tests revealed statistically significant differences between the combined PMS/PMDD group and the healthy control group in three key measures: (1) PMS severity (assessed by the PSST): PMS/PMDD group: *M* = 31.57, SD = 7.98; healthy control group: *M* = 11.14, SD = 7.90; *t*(1364) = 35.058, *p* < 0.001; (2) PHQ scores: PMS/PMDD group: *M* = 8.93, SD = 5.38; healthy control group: *M* = 3.88, SD = 3.88; *t*(1364) = 16.49, *p* < 0.001; and (3) GAD scores: PMS /PMDD group: *M* = 6.68, SD = 5.03; healthy control group: *M* = 2.39, SD = 3.31; *t*(1364) = 16.02, *p* < 0.001.

In addition, Pearson correlation coefficients between the PSST total scores, its Dimension and Impact subscale scores, PHQ scores, and GAD scores are summarized in Table 3.

**TABLE 3:** Correlations between PSST total and subscale scores, PHQ scores, and GAD scores (*n* = 1366).

	PSST total score	PSST Symptom Dimension	PSST Impact Dimension
PSST	1	0.984^**^	0.868^**^
PHQ	0.645^**^	0.632^**^	0.568^**^
GAD	0.614^**^	0.596^**^	0.555^**^

**p < 0.01.

#### 3.3.3 Construct Validity

CFA was performed to examine the factor structure of the Chinese version of the PSST, as hypothesized in the original scale development. Results indicated that the two‐factor model (Symptom Dimension and Impact Dimension) yielded acceptable to good fit indices: *χ* ^2^/df = 4.21, GFI = 0.932, CFI = 0.925, TLI = 0.905, RMSEA = 0.05. The fit of this two‐factor model was significantly superior to that of the one‐factor model (*χ* ^2^/df = 5.88, GFI = 0.795, CFI = 0.875, TLI = 0.752, RMSEA = 0.09). A chi‐square difference test for nested models confirmed that the improvement in model fit associated with the two‐factor structure was statistically significant (Δ*χ* ^2^ = 123.776, Δdf = 1, *p* < 0.001). These findings collectively support that the two‐factor structure of the PSST is more consistent with the data from the current sample.

## 4 Discussion

This study evaluated the psychometric properties of the Chinese version of the PSST among Chinese female college students, with findings confirming its satisfactory reliability and validity for PMS screening in this population.

### 4.1 Psychometric Properties of the Chinese PSST

The Chinese version of the PSST demonstrated excellent reliability: the Cronbach's *α* coefficient for the total scale was 0.91, and the test–retest reliability (ICC) reached 0.94. These values are consistent with the original scale (Steiner et al. 2003) and international adaptations, including the Iranian version (*α* = 0.93; Hariri et al. 2013), Brazilian version (*α* = 0.91; Câmara et al. 2017), and Turkish version (*α* = 0.92, ICC = 0.85; Gürses et al. 2020). Such consistency indicates that the Chinese version of the PSST maintains robust internal consistency (reflecting item homogeneity) and temporal stability (ensuring consistent results across repeated measurements), which are critical for reliable screening tools.

In terms of validity, the scale exhibited strong empirical, criterion, and construct validity. For empirical validity, participants in the PMS group and the PMDD group consistently reported higher proportions of “moderate” (score = 2) and “very severe” (score = 3) responses across all items compared to the healthy control group (all *p* < 0.001). This aligns with the original scale's development findings (Steiner et al. 2003), confirming the Chinese version's ability to distinguish between individuals with and without clinically relevant premenstrual symptoms. For criterion validity, independent samples *t*‐tests revealed that the combined PMS/PMDD group scored significantly higher on PHQ and GAD than the healthy control group (both *p* < 0.001), and Pearson correlations indicated positive associations between PSST scores (total scale and subscales) and scores on these two criterion measures. These results are consistent with the well‐established comorbidity between PMS/PMDD and depressive/anxiety symptoms (ACOG 2022), further validating the PSST's ability to capture clinically meaningful symptom severity. For construct validity, CFA showed that the two‐factor model (Symptom Dimension and Impact Dimension) yielded acceptable‐to‐good fit indices (*χ* ^2^/df = 4.21, GFI = 0.932, CFI = 0.925, TLI = 0.905, RMSEA = 0.05) and was significantly superior to the one‐factor model (Δ*χ* ^2^ = 123.776, Δdf = 1, *p* < 0.001). This supports the structural equivalence of the Chinese version to the original scale, indicating that the latent constructs of “symptom severity” and “functional impact” are applicable to Chinese college students.

### 4.2 Comparison of PMS Detection Rates

The present study reported a PMS detection rate of 14.35% and PMDD detection rate of 1.76% among female college students in Shanghai, which is lower than those reported in previous domestic studies. For example, Yuan et al. (2016) used an ACOG‐based scale and found a 24.82% combined PMS and PMDD incidence rate among 780 female college students in Xiamen; Z. Z. Zhang and Song (2010) reported a 45.4% rate (including mild symptoms) using a scale based on John's theoretical framework among 845 students in Qingdao; and Yu et al. (2016) found that 99.99% of 856 students in Jinzhou reported at least mild PMS symptoms (69.29% mild, 24.29% moderate, 6.43% severe) using the same scale as Zhang and Song.

Several factors may explain this discrepancy. First, differences in diagnostic criteria and assessment tools: The current study used the PSST, a validated screening tool focusing on moderate‐to‐severe symptoms, whereas previous studies either adopted broader diagnostic frameworks (e.g., including mild symptoms) or nonstandardized scales, leading to higher reported rates. Second, methodological variations: Variations in sample size, sampling strategies (e.g., single‐university vs. multi‐university samples), and data analysis thresholds (e.g., inclusion of mild symptoms) may contribute to inconsistent results. Third, regional disparities in mental health resources: Shanghai's universities typically offer more comprehensive mental health education and support services, which may enhance students’ awareness of PMS and PMDD management and reduce the reporting of severe symptoms (or improve coping strategies, mitigating symptom severity). Notably, this is the first study to report PMS/PMDD detection rates among female college students in Shanghai, providing a valuable regional reference for future epidemiological research.

### 4.3 Strengths and Limitations

A key strength of this study is the rigorous psychometric evaluation of the Chinese version of the PSST, including internal consistency, test–retest reliability, and multiple validity indicators, with results consistent with international adaptations. The use of two independent samples (Sample 1 for validity testing, Sample 2 for test–retest reliability) enhances the generalizability of the findings. Additionally, the focus on Chinese college students fills a gap in existing literature, as most previous adaptations targeted Western or Middle Eastern populations.

However, several limitations should be acknowledged. First, the sample was limited to female college students in Shanghai, which may limit the generalizability of the results to other populations (e.g., adolescents, perimenopausal women, or students from rural areas). Second, the cross‐sectional design prevents causal inferences about the relationship between PMS symptoms and psychological distress (e.g., depression/anxiety). Third, the PSST's cutoff value (≥10 points) was adopted from previous Chinese studies without calibration for the college student population, potentially affecting screening accuracy.

### 4.4 Future Research Directions

Based on the findings and limitations, future research should focus on the following areas:

#### 4.4.1 Cross‐Cultural Adaptation and Measurement Invariance

Future studies should conduct cross‐cultural comparisons of the PSST's dimensional structure (e.g., Chinese vs. Turkish, Brazilian, or Iranian versions) to explore cross‐cultural differences in item discriminant validity, particularly for the Impact Dimension. For example, “family function impact” may be more salient among Chinese students, whereas “occupational/academic function impact” may be emphasized in Western populations. Multi‐Group Confirmatory Factor Analysis (MG‐CFA) should be used to test the scale's measurement invariance across cultures, clarifying whether items function similarly across diverse populations and identifying culture‐specific adjustments (e.g., modifying or adding items to reflect cultural values).

#### 4.4.2 Culturally Specific Symptom Optimization

Domestic studies have identified high‐prevalence PMS symptoms among Eastern women, such as low back pain, dizziness, and fatigue (Li et al. 2021), which may be underrepresented in the original PSST. Future adaptations should conduct culture‐specific symptom analyses, supplementing or adjusting item weights to enhance coverage of region‐specific symptoms. This will reduce screening biases caused by cultural differences and improve the scale's ecological validity for Chinese populations.

#### 4.4.3 Population‐Specific Cutoff Value Calibration

Most Chinese adaptations of the PSST use a universal cutoff value of ≥10 points, which has not been validated for specific subgroups (e.g., adolescents, perimenopausal women, or clinical populations). Receiver operating characteristic (ROC) curve analysis should be used to determine optimal cutoff values for different populations, with sensitivity (≥80%) and specificity (≥75%) as key performance indicators. This will improve the scale's clinical utility for targeted screening and diagnosis.

### 4.5 Conclusion

In summary, the Chinese version of the PSST exhibits excellent reliability and validity among Chinese female college students, making it a suitable tool for large‐scale PMS screening. The findings provide empirical support for the scale's application in clinical and research settings, while identifying directions for future optimization to enhance its cross‐cultural adaptability and population‐specific utility.

## Author Contributions

**Xiaochun Wang**: writing – review and editing, project administration. **Xue Li**: investigation, writing – original draft.

## Conflicts of Interest

The authors declare no conflicts of interest.

## Funding

The authors have nothing to report.

## Consent

All female university students participating in this study have consented to complete the questionnaire and agreed to the publication of the relevant findings of this study.

## Data Availability

The data that support the findings of the study are available from the corresponding author upon reasonable request.
